# Rodent-borne viruses survey in rural settlers from Central Brazil

**DOI:** 10.1590/0074-02760180448

**Published:** 2018-12-17

**Authors:** Jorlan Fernandes, Renata Carvalho de Oliveira, Thayssa Alves Coelho, Regina Maria Bringel Martins, Karlla Antonieta Amorim Caetano, Marco Aurélio Pereira Horta, Silvana Levis, Megmar Aparecida dos Santos Carneiro, Sheila A Teles, Elba Regina Sampaio de Lemos

**Affiliations:** 1 Fundação Oswaldo Cruz-Fiocruz, Instituto Oswaldo Cruz, Laboratório de Hantaviroses e Rickettsioses, Rio de Janeiro, RJ, Brasil; 2 Universidade Federal de Goiás, Instituto de Patologia Tropical e Saúde Pública, Goiânia, GO, Brasil; 3 Universidade Federal de Goiás, Faculdade de Enfermagem, Goiânia, GO, Brasil; 4 Fundação Oswaldo Cruz-Fiocruz, Instituto Oswaldo Cruz, Rio de Janeiro, RJ, Brasil; 5 Instituto Nacional de Enfermedades Virales Humanas, Pergamino, Argentina

**Keywords:** hantavirus, arenavirus, rural settlers, rodent-borne diseases

## Abstract

Anthropogenic environmental changes arising from settlement and agriculture include deforestation and replacement of natural vegetation by crops providing opportunities for pathogen spillover from animals to humans. This study aimed to investigate the prevalence of rodent-borne virus infections in seven rural settlements from Midwestern Brazil. Of the 466 individuals tested 12 (2.57%) were reactive for orthohantavirus and 3 (0.64%) for mammarenavirus. These rural settlers lived under unfavorable infrastructure, socioeconomic disadvantages, and unsanitary conditions, representing a risk for rodent-borne infections. Development of public policies towards the improvement of health, sanitation and awareness of rodent-borne diseases in improvised camps and settlements is imperative, in order to reduce morbidity and mortality caused by these diseases.

Human behavior, especially spatial expansion of agriculture, has been implicated as drivers of some recent emerging disease events that had important impact on human health.^1^ Anthropogenic environmental changes caused by settlement and agriculture include habitat deforestation, fragmentation, and replacement of natural vegetation by crop fields.^2^ These changes modify wildlife population structure and migration and reduce biodiversity by creating environments that favor particular hosts, vectors, and/or pathogens.^1^ ^,^ ^2^

Rural settlers are a growing population in Brazil, where currently 9,256 rural settlements are listed, occupying an area of 88,314,857 hectares (three times the size of the United Kingdom).^3^ These settlements were established to encourage landless rural workers on unproductive properties to be relocate to potentially productive areas through a system of land grants. Often, before they own the land, they live in camps, where living conditions are unfavorable.^4^ In addition, many families live in rudimentary housing, devoid of piped water, electricity, selective garbage collection, conditions that favors proliferation and increases chances of contact with rodents.^4^ ^,^ ^5^ ^,^ ^6^ In this context of social vulnerability and environmental degradation, this study aimed to investigate the prevalence of rodent-borne viruses (hantaviruses and mammarenaviruses) infections in rural settlers from Brazil.

Human infections caused by hantaviruses and mammarenaviruses are associated with the natural cycle of these viruses, and transmission usually occurs by inhalation of aerosolised rodent excreta.^7^ Brazil occupies most of the eastern portion of South America, supporting several biomes with multiple natural ecosystems that account for the reported regional differences and temporal trends of hantavirus pulmonary syndrome (HPS).^8^ To date, more than 2,000 HPS cases have been reported and six hantavirus genotypes have been identified as pathogenic to humans in Brazil: Juquitiba, Araraquara, Castelo dos Sonhos, Laguna Negra, Anajatuba and Rio Mamore viruses. These viruses are transmitted by wild rodent reservoirs of the species *Oligoryzomys nigripes*, *Necromys lasiurus*, *Oligoryzomys utiaritensis*, *Calomys callidus*, *Oligoryzomys mattogrossae* and *Oligoryzomys microtis*, respectively.^7^ ^,^ ^8^ In contrast, only one case of Brazilian haemorrhagic fever was described in 1990, which was caused by Sabiá mammarenavirus, for which the animal reservoir is still unknown.^9^

A cross-sectional study was conducted in rural settlements from southwest of Goiás state, Central Brazil. Individuals from seven rural settlement projects established in Perolândia (Lagoa do Bonfim and Três Pontes) and Jataí (Santa Rita, Rio Claro, Nossa Senhora de Guadalupe, Terra e Liberdade and Gurita) counties (Figure) were interviewed using a standard questionnaire to record their sociodemographic and behavioral characteristics, as reported previously.^4^ ^,^ ^5^ Blood was collected (10 mL) from all participants, and serum samples were tested by an enzyme-linked immunosorbent assay (ELISA) for the detection of anti-hantavirus and mammarenavirus immunoglobulin G (IgG).^10^ ^,^ ^11^ This study was aproved by the Research and Ethics Committee of Hospital das Clínicas, Universidade Federal de Goiás number 127/2010, and by Fundação Oswaldo Cruz/Instituto Oswaldo Cruz Ethical Committee, number CAAE 61629416.2.1001.5248.

Briefly, 96-round-bottom-well-microplates (Thermo Scientific^TM^) were coated with 100 µL of the cell lysate diluted in phosphate-buffered saline (PBS) pH 7.4. One-half of the plate with the infected cell lysate (Junín mammarenavirus strain XJC13 or Maciel orthohantavirus strain #9) and the other half with the uninfected cell lysate (Vero C76- ATCC^®^ CRL-1587™). The plates were kept overnight at 4ºC, and then washed five times with 0.1% Tween 20 (Merck & Co., Inc., Kenilworth, NJ, USA) in PBS. The wells were then filled with 100 µL diluted test sera, starting at 1:100 dilution. As diluent, PBS with 0.1% Tween 20 (Merck & Co., Inc., Kenilworth, NJ, USA) and 5.0% skim milk (BD Difco™) was used. The plates were incubated for 1 h at 37ºC. After five washes, 100 µL of goat Anti-Human IgG peroxidase (Kirkegaard & Perry Laboratories- KPL, Gaithersburg, MD) conjugated at 1:2000 dilution was placed in each well and the plates were incubated for 1 h at 37ºC. The five washes were repeated and 100 µL of ABTS [2, 2’-azino-di (3-ethylbenzthiazoline-6-sulfonate)] substrate (KPL, Gaithersburg, MD) was added to each well. The plates were kept for 30 min at 37ºC. Objective reading of ELISA results was performed by determination of absorbencies at 405 and 450 nm. The cut-off was determined by the mean optical densities (OD) of the negative controls plus three standard deviations at 1:100 dilution.

**Map of:**
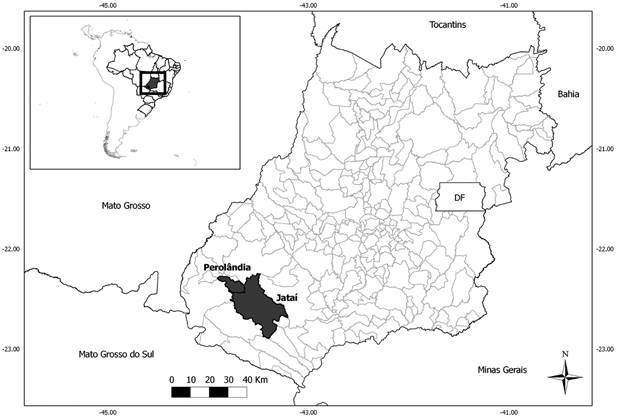
Goiás state, Midwestern Brazil, showing the municipalities of Jataí and Perolândia.

Aiming to test the association among variables, chi-square tests (χ^2^) were used to verify those variables that presented a relationship with the outcome variable (detection of anti-hantavirus IgG antibodies) in order to perform a modeling process. Univariate logistic regression analyses were performed between serology results and those other variables that demonstrated association with the outcome by χ² analysis with p-value under 30% and Odds ratios (OR) and 95% confidence intervals (95%CI) were estimated. Data analysis was performed using the statistical package R (version 3.1.1).

Nearly half of the population was male (52.1%). The median age was 41 years (minimum: 2; maximum: 93), and monthly income of approximately $210.00 USD (minimum: 39; maximum: 3460, USD, approximately). Most are self-declared mixed race (60.1%) and had a maximum of nine years of formal schooling (82.5%). About 68.0% of all participants had lived in canvas or improvised plastic tents in provisory settlements (Table).

Of the 466 individuals enrolled in the study, 12 (2.57%) were reactive for orthohantavirus and 3 (0.64%) for mammarenavirus. One individual was reactive for both tests, but high titers for orthohantavirus (1:6400) were found. Arenavirus antibodies were found only in Terra e Liberdade settlement (3/141 - 2.1%) in both sex (two men and one woman) with ages ranging from 16 to 66 years. Although we do not have detailed information on the medical history of these individuals, all of them reported having been hospitalised at some point for not specified clinical (not chirurgical) reasons.

Hantavirus antibodies were present in all seven settlements, with prevalence ranging from 0.9% (1/101 - Nossa Senhora de Guadalupe), 1.4% (2/141 - Terra e Liberdade), 2.0% (1/47 - Santa Rita), 4.0% (1/25 - Lagoa do Bonfim), 4.3% (4/92 - Gurita) and Rio Claro (1/23) to 5.4% (2/37 - Três Pontes). Six individuals referring to hospitalisation for not specified clinical reasons at some point of their lives. There was no statistically significant difference between the evidence of hantavirus infection and the variables showed in Table. Due to low number of mammarenavirus reactive samples, statistical analyses were performed considering only hantavirus reactivity.

**TABLE Distribution:** of caracteristic, prevalence of seropositivity (detection of anti-hantavirus IgG antibodies) and chi-square test (p-value) for rural population in Goiás, Brazil

Variable	N (%)	Seropositivity (%)	χ^2^ p-value	OR (95%CI)
Age			0.29	
≤ 18	118 (25.3)	1 (0.8)		1
19-40	110 (23.6)	2 (1.8)		2.16 (0.19-24.2)
41-60	191 (41.0)	8 (4.2)		5.11 (0.63-41.4)
> 61	47 (10.1)	1 (2.1)		2.54 (0.15-41.4)
Sex			0.88	
Female	223 (47.9)	6 (2.7)		
Male	243 (52.1)	6 (2.5)		
Setllement			0.59	
Terra	141 (30.3)	2 (1.4)		
Guadalupe	101 (21.7)	1 (1.0)		
Gurita	92 (19.7)	4 (4.3)		
Santa Rita	47 (10.1)	1 (2.1)		
Três Pontes	37 (7.9)	2 (5.4)		
Rio Claro	23 (4.9)	1 (4.3)		
Lagoa	25 (5.4)	1 (4.0)		
Monthly income (USD)			0.75	
≤ 330	325 (72.9)	9 (2.8)		
331-660	113 (25.3)	2 (1.8)		
≥ 661	8 (1.8)	0 (0.0)		
School (years)			0.15	
≤ 4	202 (46.3)	8 (4.0)		1
5-9	170 (39.0)	3 (1.8)		0.43 (0.10-2.17)
≥ 10	64 (14.7)	0 (0.0)		-
Smoke			0.18	
No	302 (75.9)	6 (2.0)		1
Yes	95 (29.9)	5 (5.3)		2.74 (0.81-9.19)
Time living in the settlement (days)			0.09	
≤ 30	85 (18.4)	2 (2.4)		1
31-60	265 (57.5)	4 (1.5)		0.63 (0.11-3.53)
≥ 61	111 (24.1)	6 (5.4)		2.37 (0.46-12.05)
Time living in camps (months)			0.36	
≤ 50	160 (51.3)	4 (2.5)		
50-100	116 (37.2)	5 (4.3)		
≥ 100	36 (11.5)	0 (0.0)		
Garbage			0.49	
Burnt	267 (59.7)	5 (1.9)		
Buried	19 (4.3)	0 (0.0)		
Collected	145 (32.4)	5 (3.4)		
Other	16 (3.6)	1 (6.2)		
Sanitation			0.47	
No	100 (21.7)	1 (1.0)		
Yes	361 (78.3)	11 (3.0)		

Encroachment of human settlements and agriculture on natural ecosystems results in expansion of transition zones between these two ecological systems, where species assemblages from different habitats can mix.^2^ ^,^ ^12^ This provides new opportunities for pathogen spillover, genetic diversification, and adaptation. Associations between disease emergence in transition areas have been suggested for several diseases, including yellow fever, HPS, Nipah virus encephalitis, rabies, cholera, leptospirosis.^7^ ^,^ ^13^ ^,^ ^14^

HPS is considered a rural disease and consequently rural dwellers as well as individuals who are often in contact with the rural area are considered as risk group. Goiás state has 98 confirmed HPS cases, since 2000.^7^ ^,^ ^8^ ^,^ ^15^ Two hantaviruses have been detected in the state, Araraquara and Juquitiba virus in *N. lasiurus* and *O. nigripis* rodents, respectively.^16^ The prevalence of anti-hantavirus IgG antibodies detected in this study is in accordance with prevalence found in different studies conducted in rural populations from Brazil (0.4% to 22.0%),^17^ ^,^ ^18^ ^,^ ^19^ ^,^ ^20^ ^,^ ^21^ ^,^ ^22^ ^,^ ^23^ but is lower than the one found for Jataí municipality 3.9% (95% CI: 2.3-5.7).^24^ According to Guzmán et al.,^11^ this difference could be explained by the lower sensitivity found in the serological testes using Maciel orthohantavirus when compared to those obtained using Araraquara orthohantavirus. Studies also show considerable serological prevalence for hantavirus in men, that seem to link hantavirus infection and field related activities. This fact was not observed in our study, probably due to the rudimentary living conditions, especially during their time in improvised camps, where man and woman could be exposed in their habitations during indoors activities, as reported in works conducted in Brazil and Chile.^19^ ^,^ ^22^ ^,^ ^25^

Mammarenavirus prevalence is variable in different endemic areas. Our results are comparable with ones found in other South American countries. In Argentina, studies demonstrated low prevalence of unapparent infections ranging from 1.93% (3/155) to 4.44% (24/540) for rural populations from Córdoba and Buenos Aires provinces, respectively.^26^ The prevalence of Guanarito virus infection among humans from Portuguesa state, Venezuela, was 2.60% (5/195), all adults living in rural areas and two had previously been reported as Venezuelan hemorrhagic fever cases.^27^ Machado et al.^28^ observed that 1.40% (5/343) individuals living in the municipality of Nova Xavantina (Mato Grosso state, Brazil), who presented fever with unidentified etiology, presented antibody titers against arenavirus. It is important to notice that Argentina and Venezuela are endemic countries for arenavirus hemorrhagic fevers, with several reported cases. Thus, during the emergence of American hemorrhagic fever a scenario of sociodemographic expansion and increased deforestation was described in Argentina and Venezuela,^26^ ^,^ ^27^ the same factors that are present in the studied area.

To date there are few evidences of arenaviruses causing human infections in Brazil; the presence of antibodies in rural population in similar rates to those found in endemic countries points out for a probable silent circulation of mammarenaviruses in rural areas of Brazil.^28^ ^,^ ^29^ The only settlement where serological evidences for arenavirus were found (Terra e Liberdade - Jataí county) is 953 km of distance from the probable local of infection of the first and only case of Brazilian hemorrhagic fever reported.^9^ To date, at least eight mammarenavirus have been described in Brazil, two from Midwestern states.^30^ ^,^ ^31^ The identification of seroreactivity in just one settlement could represent a local circulation of a distinct mammarenavirus, especially considering the natural nidality attributed to some mammarenaviruses.^32^

Until Brazilian government established these settlements between 1998 and 2007, many of the landless rural residents lived in this precarious condition, arranging in canvas or plastic tents, for many years. The long period under unfavorable infrastructure, socioeconomic disadvantages, and unsanitary conditions may represent a risk of acquiring hantavirus and mammarenavirus infections. The intense process of degradation in recent decades, with large areas planted with monoculture crops, can favor generalist rodent species in interspecific competition with other rodents, mostly habitat specialists. The general behavior of many rodent-borne reservoirs as *N. lasiurus* and *O. nigripes* make these species highly adaptable to man-made environments.^16^ The ecological pattern of these rodents associated with deep anthropogenic changes areas provide by improvised camps or rural settlements greatly increases the chances of contact between humans and infected rodents, mainly due to the potential increase in population density and the narrowing of environments shared with humans.^2^ ^,^ ^6^ ^,^ ^16^

Rodent-borne infections are highly lethal diseases especially in rural areas, where poor people that depend on the land to survive are constantly exposed to rodents, with low access to medical care. Implementation of public policies towards the improvement of health access, sanitation and awareness of rodent-borne diseases in improvised camps and settlements is needed, in order to prevent human infection and reduce morbidity and mortality caused by these diseases. In the future, sustainable agricultural systems that minimise the risk of emerging disease will therefore be essential to meet the requirements of the rising rural settlers’ population, while protecting human health and conserving biodiversity and the environment.
